# Occurrence of Free and Conjugated Mycotoxins in Aromatic and Medicinal Plants and Dietary Exposure Assessment in the Moroccan Population

**DOI:** 10.3390/toxins13020125

**Published:** 2021-02-08

**Authors:** Aicha El Jai, Abdellah Zinedine, Ana Juan-García, Jordi Mañes, Samira Etahiri, Cristina Juan

**Affiliations:** 1 Laboratory of Marine Biotechnologies and Environment (BIOMARE), Faculty of Sciences, Chouaib Doukkali University, P.O. Box. 20, El Jadida 24000, Morocco; aichaeljai01@gmail.com (A.E.J.); setahiri@hotmail.com (S.E.); 2 Laboratory of Food Chemistry and Toxicology, Faculty of Pharmacy, University of Valencia, E-46100 Valencia, Spain; ana.juan@uv.es (A.J.-G.); jordi.manes@uv.es (J.M.); cristina.juan@uv.es (C.J.)

**Keywords:** mycotoxins, co-occurrence, Q-TOF-LC/MS, exposure, Morocco

## Abstract

Aromatic and medicinal plants (AMPs), as herbal material, are subjected to contamination by various mycotoxin-producing fungi, either free and conjugated. Such a problem is associated with poor storage practices, and lack of adopting good agricultural practices and good harvesting practices. Nevertheless, AMPs are poorly investigated. The purpose of this study was to investigate the co-occurrence of 15 mycotoxins (four aflatoxins (AFB1, AFB2, AFG1, and AFG2), ochratoxin A (OTA), beauvericin (BEA), four enniatins (ENA, ENA1, ENB, and ENB1), zearalenone (ZEN), alternariol (AOH), tentoxin (TENT), T-2, and HT-2 toxins) in 40 samples of AMPs frequently consumed in Morocco by using liquid chromatography tandem mass spectrometry. Evaluation of conjugated mycotoxins and their identification using liquid chromatography coupled to time-of-flight mass spectrometry with ion mass exact was also carried out. Results showed that 90% of the analyzed samples presented at least one mycotoxin, and 52% presented co-occurrence of them. Mycotoxins detected were: AOH (85%), ZEN (27.5%), β-ZEL (22%), AFG1 (17.5%), TENT (17.5%), ENB (10%), AFG2 (7.5%), α-ZEL (5%), ENA1 (2.5%), and HT-2 (2.5%), while the conjugated mycotoxins were ZEN-14-Glc (11%) and ZEN-14-Sulf (9%). The highest observed level was for AOH, with 309 ng/g. Ten samples exceeded the recommended levels set by the European Pharmacopoeia for AF mycotoxins in plant material (4 ng/g), and three samples exceeded the maximum limits for AFs (10 ng/g) in species established by the European Commission. Although the co-occurrence of several mycotoxins in AMP samples was observed, the dietary exposure assessment showed that the intake of mycotoxins through the consumption of AMP beverages does not represent a risk for the population.

## 1. Introduction

Aromatic and medicinal plants (AMPs) are known to contain various compounds that can be valorized for several purposes, including preservative, therapeutic, and organoleptic proprieties, most of which are precursors for chemo-pharmaceutical semi-synthesis [1,2]. AMPs have been widely used to treat and/or prevent diseases and promote health since ancient times. However, AMPs are frequently exposed to various fungi responsible for the decrease in market quality, as their growth allows the presence of mycotoxins. Such fungi respond to contamination in soil, or during harvesting, drying, transport, manipulation, or storage [3,4].

Nowadays, several studies have reported the presence of mycotoxins in herbal plants and derivative products, considering that this contamination is a global issue, particularly in developing countries [5]. In fact, previous investigations reported that, under specific conditions, toxigenic fungal species from *Aspergillus, Penicillium, Fusarium,* and *Alternaria* genus can generate mycotoxins and contaminate herbal medicines. Indeed, these reports described mycotoxin contamination of medicinal herbs and related products, showing that mycotoxins, such as aflatoxins (AFs), ochratoxin A (OTA), zearalenone (ZEN), fumonisins, and trichothecenes are the most commonly present [6]. Another important point is related to the metabolization that can partially suffer from some mycotoxins, such as ZEN and deoxynivalenol (DON), by the fungus producer and by the infected host plant. The Phase I and II reactions in the metabolism process aim to eliminate these compounds, and this is often accomplished by the attachment of hydrophilic groups. The most indicated enzymatic system in the literature is the UDP-glucosyltransferase (UGT), which is capable of converting ZEN into ZEN-4-glucoside (ZEN-4-Glc) [7,8,9], or in Zearalenone-14-Glucoside (ZEN-14-Glc) and Zearalenone-16-Glucoside (ZEN-16-Glc) [10]. Furthermore, α-zearalenol (α-ZEL) and β-zearalenol (β-ZEL) suffer glycosylation as part of the host plant’s metabolism, leading to α–ZEL-14-glucoside (α-ZEL-14-Glc) and β–ZEL-14-glucoside (β-ZEL-14-Glc), respectively [11]. This happens with other mycotoxins, such as DON, which is converted to DON-3-glucoside (DON-3-Glc) [12,13]. Moreover, during fungal metabolism, the sulfate form of ZEN, which is partially converted to Zearalenone-4-sulfate (ZEN-4-Sulf), has been found [14,15]. Despite their chemical alteration, there is evidence that metabolites have a similar toxic potential to those of their precursors when it is ingested, as attached functional groups like glycosylic or sulfate residues are likely to be enzymatically cleaved during digestion [14,16]. In most of the analytical approaches published, the main target of study has been the parental molecules forgetting the detection of their metabolites, resulting in an underestimation of the inherent toxicity of a contaminated sample. Altered metabolites of mycotoxins are also referred to as masked mycotoxins [16], thus, it is important to perform a non-target analysis to study the formation, determination, and significance of masked and other conjugated mycotoxins present in foodstuffs [17].

There is legislation established for mycotoxins in some spices and plants to avoid hazardous effects associated with their presence in herbal material [18]. The European Commission has established maximum limits (MLs) for mycotoxins, such as AFB1 (5 ng/g) and AFs (10 ng/g) in various spices (*Capsicum spp., Piper spp., Myristica fragrans, Zingiber officinale,* and *Curcuma longa*); however, no MLs have been established for aromatic plants [19]. In the European Pharmacopoeia (2016) [20], MLs have been set for AFs as 2 ng/g for AFB1 in herbal drugs and 4 ng/g for the sum of AFs [21]; while in the US Pharmacopeia (USP), ML of 5 ng/g for AFB1 and 20 ng/g for AFs have been implemented for certain types of raw medicinal herb materials, as well as their by-products (in powder and/or dry extract) [6]. 

In Morocco, several reports have evaluated the presence of mycotoxins in spices and herbal materials [22,23,24]. Recent Moroccan regulations have set MLs for certain mycotoxins in food products. For example, the ML is set at 5 ng/g for AFB1, 10 ng/g for the sum of AFs, and 15 ng/g for OTA in selected spices, such as *Capsicum spp., Piper spp., Myristica fragrans, Zingiber officinale,* and *Curcuma longa* [25]. However, no information is available regarding the possible co-occurrence of mycotoxins in AMP samples consumed in the country. Thus, the aims of the present study were: (i) to develop a liquid chromatography tandem mass spectrometry (LC-MS/MS) method to determinate 15 mycotoxins in AMPs from Morocco; (ii) to develop a liquid chromatography coupled with a time-of-flight mass spectrometry (LC-QTOF-MS) method as the screening tool to obtain not only confirmation, but also the detection of possible co-occurrence non-target mycotoxins, including masked mycotoxins; and (iii) to apply the developed method in the most consumed Moroccan AMP varieties supplied from the Moroccan market to investigating their co-occurrence. Data obtained from contamination levels will permit the assessment of the risk of dietary exposure for the Moroccan population to these mycotoxins through AMP intake.

## 2. Results and Discussion

### 2.1. Validation

Mycotoxin analysis in food and plant ingredients is always based on several factors, including the composition and nature of the matrix or ingredients to control, and the mycotoxin to investigate. In this study, the used modified dispersive liquid−liquid microextraction (DLLME) and LC-MS/MS methods were checked, and validation of the parameters of recoveries, linearity, LOD, and LOQ were carried out (Table 1 and Supplementary Table S1).

Linearity was evaluated with calibration curves, which were constructed for each mycotoxin with methanol and a blank sample at concentration levels ranging from the LOQ to 1 μg/mL. All studied mycotoxins presented good linearity, with correlation coefficients (r^2^) higher than 0.9995. Substances present in the matrix that modified the instrumental response of the analyte were evaluated by matrix effects (MEs), resulting in enhancement or suppression of the signal, so that signal suppression/enhancement was evaluated comparing the slopes of calibration curves obtained with methanol and in the blank sample. ME values higher than 100% indicated enhancement of the signal, ME values lower than 100% indicated suppression of the signal, and ME values near 100% indicated no significant matrix effects. The accuracy has been evaluated with recoveries (R) of the analytical method, so that a mix of blank samples (negative, < LOD) were spiked at three levels (LOQ, 2 LOQ. and 10 LOQ). Recovery values ranged from 81 ± 4.26% (HT-2) to 125 ± 14.18% (EN A1) at 10 times LOQ (Table 2). In Supplementary Table S2, the R at 10 times LOQ (*n* = 3, intraday study) and the ME observed by each studied AMP sample (*n* = 3) are shown. High ion suppression in *Rosmarinus officinalis, Matricaria chamomilla,* and *Myrtus communis* samples were observed.

### 2.2. Natural Occurrence of Mycotoxins

Out of 40 total AMP samples, 36 samples (90%) presented at least one mycotoxin (Table 3). All analyzed samples of *Mentha spicata, Lavandula intermedia, Matricaria chamomilla,* and *Myrtus communis* were contaminated by at least one mycotoxin. The most frequent mycotoxins in AMP were AOH (85%), ZEN (27.5%), AFG1 (17.5%), TENT (17.5%), ENB (10%), AFG2 (7.5%), ENA1 (2.5%), and HT-2 (2.5%) (Table 3), while the mycotoxins AFB1, AFB2, OTA, BEA, ENA, ENB1, and T-2 were under the LOD. The highest mycotoxin value found in AMP samples was registered in a sample of *Origanum vulgare,* with 309 ng/g of AOH (Table 3). Below, the occurrence in the analyzed samples by group of mycotoxins is detailed.

Raw tea and herbal infusion materials were reported to contain up to 76 μg/kg of fumonisin B1, but no mycotoxins were detected in infusions [25]. In China, the presence of ZEN and its metabolite α-zearalenol (α-ZEL) in 100 widely-consumed foods and medicinal plants was investigated. Authors reported that 12% of these tested samples were contaminated with ZEN at levels ranging from 5.3 to 295.8 μg/kg [26]. Another study from Spain reported the occurrence of T-2 and HT-2 in seeds of milk thistle (*Silybum marianum*) at levels ranging from 363 to 453.9 μg/kg and from 826.9 to 943.7 μg/kg, respectively [27]. A study from India reported that dried market samples of stem portions of *Tinospora cordifolia,* an important medicinal plant, were contaminated with AFB1, AFB2, OTA, patulin, and citrinine; however, fusarial species and their toxins were not detected in those samples [28]. In Latvia, the occurrence of 12 mycotoxins has been recently investigated in 60 herbal teas. Among the dry tea samples, 90% were positive from one to eight mycotoxins. ENB, DON, AFB1, and OTA were the most frequently detected mycotoxins in 55%, 45%, 20%, and 10% of samples, respectively. The authors reported that 32% and 100% of DON and ZEN, respectively, present in dry teas were extracted into the infusions ready for its consumption [29]. A study from Spain showed the presence of AFB2 (19.1–134.7 μg/L) and AFG2 (2.2 to 13.5 μg/L) in botanical dietary supplement infusion beverages, and ENB in two samples, although at low levels [30]. More recently, AFs were detected in green tea samples obtained from retail shops and supermarkets in three Moroccan areas; however, the rate transfer of AFs from herbal green tea to infusion was unavailable, as it was not investigated [31].

#### 2.2.1. Aflatoxins (AFG1 and AFG2)

AFG1 was detected in seven samples (17.5%), including two samples of *Lavandula intermedia* (5%) and five samples of *Myrtus communis* (12.5%) (Table 3 and Table 4). Levels of AFG1 ranged from 4.9 to 8.6 ng/g, and the mean level of AFG1 in positive samples was 4.6 ± 1.4 ng/g (Table 4). Concerning the presence of AFG2, it was detected in three samples of *Rosmarinus officinalis* (7.5%). Levels of the AFG2 in this plant ranged from 26.2 to 41.1 ng/g, with a mean value of 27.7 ± 2.1 ng/g (Table 3 and Table 4). It should be highlighted that three positive AMP samples (*Lavandula intermedia*, *Myrtus communis,* and *Rosmarinus officinalis)* exceeded the ML (10 ng/g) of Moroccan regulation set for the sum of AFs [32].

#### 2.2.2. *Fusarium* Toxins (ZEN and HT-2)

Determination of ZEN in AMP samples showed that 27.5% of samples were positive for this mycotoxin (Table 4) specifically: two samples of *Mentha spicata*, six samples of *Rosmarinus officinalis,* and three samples of *Origanum vulgare*. Levels of ZEN varied between 33.7 and 114.7 ng/g, and the mean ZEN level was 55.7 ± 26 ng/g. Recent studies have also detected ZEN in AMP as Duarte et al., who detected them in 19 herb samples with smaller ranged values (1.82–19.02 ng/g) than those detected in our analyzed samples [33]. Concerning the presence of HT-2, one AMP sample of *Verveine officinale* (2.5%) contained this mycotoxin, with levels up to 2.9 ng/g, and a mean level of 1.47 ± 2.6 ng/g.

#### 2.2.3. Emerging Mycotoxins (ENA1 and ENB)

In this survey, only ENA1 and ENB were detected among emerging mycotoxins in AMP samples. Regarding the occurrence of ENA1 in AMP samples, this toxin was detected only in one sample (2.5%) of *Verveine officinale,* with a contamination level up to 0.3 ng/g and a mean level of 0.16 ± 0.3 ng/g. For the presence of ENB in AMP samples, four samples (10%) were contaminated: one sample of *Verveine officinale* and three samples of *Lavandula intermedia* (Table 3). Levels of the ENB were detected in a range of 0.04–0.1 ng/g, and the mean level was 0.05 ± 0.1 ng/g. 

#### 2.2.4. *Alternaria* Toxins (AOH and TENT)

*Alternaria* mycotoxins gain more and more interest due to their frequent contamination of food commodities. Indeed, these toxins are often detected in fruits, vegetables, and wines [34]. Besides the estrogenic activity demonstrated in vitro for certain *Alternaria* toxins, AOH causes DNA damage and cell cycle arrest [35]. In the present survey, 34 samples (85%) presented levels of AOH. Nine of them were *Origanum vulgare.* Levels of AOH ranged from 2.3 to 309 ng/g, and the mean level was 126.2 ± 40.4 ng/g. TENT was detected in seven samples (17.5%) as follows: five samples of *Myrtus communis* and two samples of *Lavandula intermedia*. Levels of TENT varied from 0.7 to 4.5 ng/g, and the mean level was 1.47 ± 0.85 ng/g.

### 2.3. Co-Occurrence of Mycotoxins in AMP 

**Figure 1:**
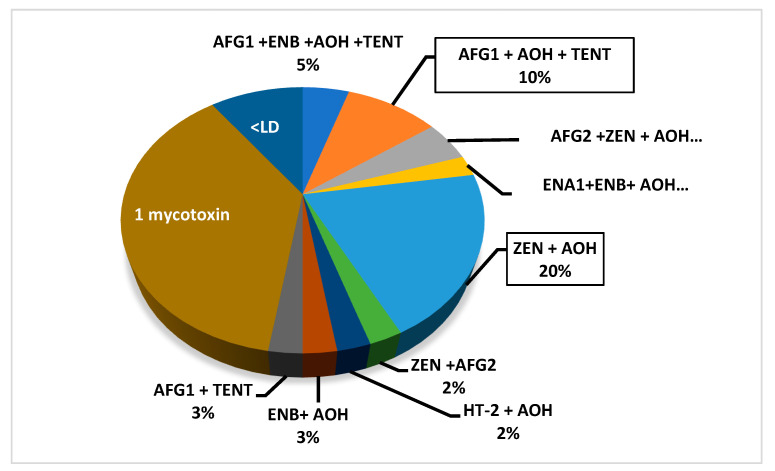
Co-occurrence mycotoxin distribution.

The co-presence of mycotoxins in a single sample could be a health concern due to the exposure of consumers to multiple fungal metabolites, which might exert greater toxicity than the exposure to a single one. The multi-mycotoxin occurrence in food and feed could be associated with health and reproductive disorders, lower performance in animals, and higher medical costs [36]. Concerning the mycotoxins’ co-occurrence in AMP samples, this happened in 52% of samples. Figure 1 summarizes the data obtained on the multi-contamination of AMP samples, revealing that the ZEN + AOH combination was the most commonly present (20%). 

Analytical results showed that four mycotoxins co-occurred in samples of *Verveine officinale* (AOH + HT-2 + ENA1 + ENB) and *Lavandula intermedia* (AFG1 + ENB + AOH + TENT), three mycotoxins were present in samples of *Rosmarinus officinalis* (AFG2 + AOH + ZEN), and *Myrtus communis* (AFG1 + AOH + TENT)*,* while two mycotoxins (AOH + ZEN) co-occurred in *Origanum vulgare* and *Mentha spicata* samples (Figure 1). Finally, positive samples of *Artemisia absinthium* and *Matricaria chamomilla* were contaminated individually by AOH.

To the best of our knowledge, limited data have been published on the multi-presence of mycotoxin in aromatic and medicinal herbs available worldwide. Indeed, a recent investigation from Spain was performed to screen the multi-contamination by mycotoxins (AFs, OTA, ZEN, T-2, DON, citrinin, and fumonisins) in 84 samples of aromatic and/or medicinal herbs, showing that 99% of the samples were contaminated with T-2 (99%), ZEN (98%), AFs (96%), OTA (63%), DON (62%), citrinin (61%), and fumonisins (13%) [37]. 

### 2.4. Conjugated Mycotoxins in AMP 

Samples with contamination levels of target mycotoxins were injected into the LC–QTOF–MS system to confirm their presence and to study the possible co-occurrence of lesser known non-target mycotoxin metabolites formed during detoxification and glycosylated and sulfated conjugates, since the probability of identifying these compounds was reasonably greater when more mycotoxins were present. 

In the present work, the exact mass and isotope pattern calculated from the molecular formula and plus/minus the expected adduct(s) of the suspected substance and experimental information (retention time behavior and presence of related substances) were used to screen that substance in the samples. Afterwards, non-target components of modified and conjugated compounds were studied to gain confidence through library match and/or diagnostic fragments.

All target mycotoxins previously quantified by LC–MS/MS were confirmed by LC–QTOF–MS (Table 4). However, the results of the study of non-target components derived from metabolism were only presented for ZEN. Although AOH was the most detected mycotoxin, ZEN was the second mycotoxin highly present in Moroccan AMP samples, and more susceptible to suffering from glycosylation, sulphuration, and hydroxylation reactions than AOH, as reported in the literature in the last decade [7,11,38]. After automatic acquisition mode MS/MS, α-ZEN, β-ZEL, ZEN-4-Glc, ZEN-14-Glc, ZEN-16-Glc, α-ZEL-14-Glc, β-ZEL-14-Glc, and ZEL-4-Sulf were selected to be identified by MassHunter Qualitative Analysis B.10.0. Mycotoxins or metabolites that have an available commercial standard (α-ZEL, β-ZEL, ZEN) were confirmed and quantified with the same program, and a “Find by Formula” data-mining algorithm with a mass error below ± 5 ppm with score values ≥ 70 (including isotope abundance and isotope spacing) was also used. A retention time window of ± 1 min was specified for peak detection to compensate for retention time shifts due to system-to-system variability. All relevant compound species including adducts: [M+H]^+^, [M+Na]^+^, [M−H]^–^, [M+HCOO]^–^, [M−OH]^–^, and [M+HCOOH−H]^–^ were used as target masses. Mycotoxin metabolites’ annotation was also supported by comparing the obtained MS/MS fragmentation spectra with the experimental spectra proposed in the databases of mycotoxins and related metabolites, Personal Compound Database and Library (PCDL) Manager MassHunter. Metlin Metabolites PCDL MassHunter contains an accurate mass compound database, a collision cross section database, and an MS/MS accurate mass spectral library for mycotoxins.

In the present study, the ZEN conjugates and metabolites selected were α-ZEL, β-ZEL, ZEN-14-Glc, β-ZEL-14-Glc, and ZEN-4-Sulf. All were selected based on purity score values ≥ 70 and a mass error < ± 5 ppm. Tentative identification is listed in Table 5. β-ZEL was the most abundant metabolite found (22%). The glycosylated ZEN (ZEN-14-Glc) compound was detected in five samples (11%), with low intensity. Regarding ZEN sulfate conjugate (ZEN-4-Sulf), it was detected in four samples (9%). Previous studies have indicated the presence of ZEN-4-Sulf in fungal cell cultures in molar ratios from 1:12 to 1:1, compared with ZEN [14], and in wheat flour samples at levels of 9.7% ZEN-4-Sulf [38]. Here, in AMP samples, ZEN-4-Sulf was present at levels ranging from 5 to 2% of the ZEN level. As far as we know, this is the first time that these non-target metabolites have been reported in AMP samples, and the first time they have been identified by LC–QTOF–MS in AMP. QTOF-MS data cannot support quantification of compounds without the use of a reference compound so that the conjugated mycotoxins detected were not quantified, although their presence was positive. Supplementary Figure S1 shows a total ion current (TIC) chromatogram from a positive sample and its extracted ion chromatogram (EIC) of β-ZEL and ZEN-14-Glc detected. Their corresponding scan and product ion spectrum are included in Supplementary Figure S1. 

### 2.5. Dietary Exposure

Several studies have reported the presence of mycotoxins in food and feed, while few data are still available of mycotoxins present in medicinal plants. The effect of mycotoxins found in some herbal plants on biochemical parameters of blood from mice were reported by Alwakeel (2009) [39]. The authors showed that analytical parameters, such as mean creatinine, urea, alanine aminotransferase, aspartate aminotransferase, and gamma-glutamyl transpeptidase, were higher in the mice group fed or treated with herbal and fungal extracts than the control group, and the study confirms the implication of the AFs with induction of nephrotoxicity and hepatoxicity in animals.

No data is currently available on the annual consumption of AMPs in Morocco, and as for estimation, it is assumed that the annual AMP consumption is half of the annual raw tea consumption. According to FAO, the annual consumption per capita of green tea in Morocco averages 1.89 kg/year, so the annual consumption per capita of AMPs in Morocco is supposed to be 0.94 kg/year [40]. The risk of mycotoxins was assessed herein following both lower bound (LB) and upper bound (UB) approaches. For the LB approach, mean values were obtained by assigning a level of zero to free mycotoxin samples (where no mycotoxins were detected), or at levels below the LOQ (where mycotoxins were detected), whereas, in the UB approach, values equal to the LOD were assigned to samples where no mycotoxins were detected, and values equal to the LOQ were assigned to samples in which mycotoxin levels were below the LOQ.

In this study, mycotoxins AFG1, AFG2, ZEN, HT-2, AOH, TENT, ENA1, and ENB were detected in positive samples. For the AFs, these substances are confirmed as carcinogenic and classified by IARC in Group 1 (do not have an established TDI), so it is not possible to determine the threshold levels at which AFs have no effect [41]. It is recommended by JECFA, with regard to the safe level of AFs in foods, that AF levels must be reduced according to the “As Low As Reasonably Achievable” (ALARA) principle [42]. Furthermore, no TDI values have been established for emerging mycotoxins (BEA and ENs) and *Alternaria* toxins (AOH and TENT), so a risk assessment is not possible to calculate for these mycotoxins. For ZEN, the PDIs calculated were 0.67 ± 1.21 ng.kg^−1^.bw.day^−1^ (LB approach) and 1.39 ± 1.20 ng.kg^−1^.bw.day^−1^ (UB approach), and the TDIs (ZEN 250 ng.kg^−1^.bw.day^−1^) were 0.56% and 0.82% by the LB and UB approaches, respectively (Table 4).

Risk assessment shows that the intake of mycotoxins through the consumption of AMP beverages does not represent a risk for the population, except for AFs that are classified as carcinogenic compounds. Nevertheless, the presence of mycotoxins in AMPs could increase the exposure in large consumers. A focus on plants is relevant to gather more knowledge on larger spectra of mycotoxin contamination related with AMP handling conditions, their presence in extracted essential oils, the apparition of any toxic effect, or the effect on human health.

Another important point is that the conjugated metabolites, as well as their reductive forms, are not a part of ZEN´s regulations. In vitro analyses of the gastrointestinal digestive process showed no cleavage of ZEN´s conjugates, but in human microbiota fermentation, the conjugates were cleaved by the microbial enzymes [43,44]. Thus, ZEN uptake might be underestimated, due to the release of absorbable ZEN.

Recently, the EU-CONTAM Panel found it appropriate to set a group TDI for ZEN and its modified forms [45]. It must be considered that the estrogenic potency of ZEN derivatives differs. Potency factors assigned to these derivatives by the EFSA CONTAM Panel are 0.2 for β-ZEL and 60 for α-ZEL relative to ZEN. Moreover, for sulfate and glucoside conjugates, the same factors as the free forms are proposed. However, to obtain more data on the occurrence of ZEN metabolites in food and feed, standard compounds are needed.

## 3. Conclusions

Mycotoxin analysis showed that 90% of Moroccan AMP samples were positive and 52% presented co-occurrence. Besides the high incidence in samples, the concentration ranged from 0.35 ng/g (ENA1) to 309 ng/g (AOH). The most detected were AOH (85%) and ZEN (27.5 %), while ZEN + AOH was the most frequent co-occurrent mycotoxins (20% of positive samples). AFs were present in 25% of samples (*Lavandula intermedia*, *Myrtus communis,* and *Rosmarinus officinalis),* seven of which exceeded the European and Moroccan recommended levels [19,20,32].

In the present study, a sensitive, rapid, robust, and reliable LC–MS/MS method was validated for the simultaneous determination of 15 target mycotoxins in five different species of AMP: *Origanum vulgare, Rosmarinus officinalis, Matricaria chamomilla, Myrtus communis,* and *Verveine officinale.* A new LC–QTOF–MS method was applied for the simultaneous screening of non-target mycotoxins and conjugated mycotoxins in positive AMP samples. ZEN-14-Glc (11%) and ZEN-14-Sulf (9%) conjugated mycotoxins were detected in AMP samples. The strategy of combining QTOF–MS and MS/MS detectors with LC is a powerful approach for the routine monitoring of mycotoxin and conjugated mycotoxins in contaminated AMPs and other foodstuffs, providing quality and safety to the food industry and consumers.

## 4. Material and Methods 

### 4.1. Chemicals and Reagents

Standards of mycotoxins (four aflatoxins (AFB1, AFB2, AFG1 and AFG2), ochratoxin A (OTA), beauvericin (BEA), four enniatins (ENA, ENA1, ENB, and ENB1), zearalenone (ZEN), α-zearalenol (α-ZEL), β-zearalenol (β-ZEL), alternariol (AOH), tentoxin (TENT), T-2, and HT-2 toxins) were purchased from Sigma Aldrich (St. Louis, MO, USA). Individual stock solutions containing a concentration of 1000 µg/mL were prepared in methanol. Working solutions were prepared starting from the appropriate individual stock solutions. All solutions were prepared and stored in the dark at −20 °C. Methanol and acetonitrile (≥ 99.9% purity) liquid chromatography tandem mass spectrometry grades (LC-MS/MS) were supplied by VWR international Eurolab (Barcelona, Spain). Formic acid (≥ 98%) was obtained from Sigma Aldrich (St. Louis, MO, USA ). Ammonium formate (≥ 99.995%) and chloroform (CHCl_3_) (99%) were obtained from Merck KGaA (Darmstadt, Germany). Ethyl acetate (EtOAc) (HPLC-grade, > 99.5%) was purchased from Alfa Aesar (Karlsruhe, Germany). The water used was purified (≤ 10 MΩ cm^−1^ resistivity) in the laboratory using a Milli-Q SP^®^ Reagent water system (Millipore, Bedford, MA, USA).

### 4.2. Plant Sampling

A total of forty AMP samples were randomly collected in 2019 from local markets (retailers and supermarkets) in three different areas of Rabat (Morocco): Témara, Bitat, and Kamra. All samples belonged to eight varieties of AMP plants (*Origanum vulgare* (*n* = 12), *Rosmarinus officinalis* (*n* = 7), *Myrtus communis* (*n* = 5), *Matricaria chamomilla* (*n* = 5), *Verveine officinale* (*n* = 4), *Mentha spicata* (*n* = 2), *Lavandula Intermedia* (*n* = 3), and *Artemisia absinthium* (*n* = 2)). The selection of these plants was based on the criteria that they are among the most traditionally used plants and consumed by the Moroccan population for their aromatic and/or therapeutic properties [46]. The amount of each AMP sample was at least 50 g, packed in bags and stored in a dark and dry place until analysis.

### 4.3. Mycotoxin Extraction Procedure

The sensitive and accurate analysis of mycotoxins in complicated matrices (e.g., herbs) typically involves challenging sample pretreatment procedures and an efficient detection instrument. A modified DLLME method [47] was applied to extract the studied mycotoxins. Firstly, 2 g of AMP samples were boiled with 200 mL of water for 5 min in a glass container. Next, 10 mL of the aqueous solution tea filtrated with Whatman filter paper was placed in a conical polytetrafluoroethyl (PTFE) centrifuge tube (15 mL), and 2 g of NaCl was added. Then, 1.9 mL of acetonitrile (dispersion solvent) and 1.24 mL of ethyl acetate (extraction solvent) was added and vortexed for 1 min. It was centrifuged for 5 min at 4000 rev/min at 5 °C, using Eppendorf centrifuge 5810R (Eppendorf, Hamburg, Germany), and a cloudy solution of the three phases was formed. The organic phase at the top (Tube 1) was recovered and placed in a second PTFE centrifuge tube (15 mL, Tube 2), while the remaining residue (Tube 1) was saved for a second extraction with 3.2 mL of a mixture of methanol and chloroform (60:40, *v*/*v*). Then, Tube 1 was vortexed for 1 min, and, with a centrifugation at 4000 rev/min for 5 min at 5 °C, the separated organic phase was recovered and added to the collected organic phase in Tube 2.

Both separated organic phases in PTFE centrifuge Tube 2 were evaporated to dryness under a nitrogen stream using a TurboVap LV evaporator (Zymark, Hopkinton, MA). The dried residue was reconstituted with 1 mL of methanol and water (70:30, *v*/*v*), and filtered through a 13 mm/0.22 μm nylon filter (Membrane Solutions, Plano, TX, USA). Next, 20 μL of the filtrate was injected into the LC-MS/MS analysis.

### 4.4. Analysis of Mycotoxins by LC-MS/MS

Analysis of the mycotoxins was performed using an LC Agilent 1200 with a binary pump and an automatic injector, and coupled to a 3200 QTRAP^®^ABSCIEX (Applied Biosystems, Foster City, CA, USA) equipped with a Turbo-V^TM^ source (ESI) interface. The chromatographic separation of the analytes was conducted at 25 °C with a reverse phase analytical column Gemini^®^ NX-C18 (3 μm, 150 × 2 mm ID) and a guard column C18 (4 × 2 mm, ID; 3 μm). The mobile phase was a time programmed gradient using water (0.1% formic acid and 5 mM of ammonium formate) as phase A, and methanol (0.1% formic acid and 5 mM of ammonium formate) as phase B [48]. The following gradient was used: equilibration for 2 min at 90% A, 80–20% A in 3 min, 20% A for 1 min, 20–10% A in 2 min, 10% A for 6 min, 10–0% A in 3 min, 100% B for 1 min, 100–50% B in 3 min, return to initial conditions in 2 min and maintain during 2 min. The flow rate was 0.25 mL/min in all steps. The total run time was 21 min.

Regarding mycotoxin analysis, the QTRAP System was used as the triple quadrupole mass spectrometry detector (MS/MS). The Turbo-V™ source was used in a positive mode to analyze the 15 mycotoxins with the following settings for source/gas parameters: Vacuum Gauge (10 × 10^−5^ Torr) 3.1, curtain gas (CUR) 20, ion spray voltage (IS) 5500, source temperature (TEM) 450 °C, and ion source gas 1 (GS1) and ion source gas 2 (GS2) 50. The fragments monitored (retention time, quantification, ion, and confirmation ion) and spectrometric parameters (de-clustering potential, collision energy, and cell exit potential) used were those performed previously (Juan et al. 2019), and are shown in Table 1.

### 4.5. Method Validation

Validation of the LC-MS/MS method was performed for linearity, repeatability (intraday and inter-day precision), and sensitivity, following the EU Commission Decision 2002/657/EC (EC, 2002). Matrix-matched calibration curves were constructed at concentration levels between the LOQ to 1 μg/mL. The matrix effect (ME) was assessed for each analyte by comparing the slope of the standard calibration curve (standard) with that of the matrix-matched calibration curve (matrix) for the same concentration levels. The limit of detection (LOD) and limit of quantification (LOQ) were estimated for a signal-to-noise ratio (S/N) ≥ 3 and ≥ 10, respectively, from chromatograms of samples spiked at the lowest level validated. LOD and LOQ values were established as a mean of the LOD and LOQ for each matrix and a mix with all studied matrixes, in this way taking into account the possible heterogeneity of the samples. Accuracy of the studied mycotoxin extraction from AMP samples was determined by a mix blank samples fortification procedure. The mix blank was prepared using each AMP sample studied (*Origanum vulgare, Rosmarinus officinalis, Matricaria chamomilla Myrtus communis, Verveine officinale*, *Mentha spicata*, *Lavandula Intermedia,* and *Artemisia absinthium*), which initially tested negative, and was fortified before the extraction procedure with three different mycotoxin levels. The concentrations of studied mycotoxins for reproducibility and repeatability studies in AMP samples were at LOQ, 2 LOQ, and 10 LOQ. Three replicates were prepared for each spiking level. Intra-day precision (repeatability) and inter-day precision (reproducibility) of the method were carried out by spiking the mix blank at the three levels previously indicated. Method precision was estimated by calculating the relative standard deviation (RSD_R_) using the results obtained during intra-day and inter-day replicate analysis (*n* = 9).

### 4.6. Analysis of Mycotoxin Metabolites by LC-QTOF-MS 

The QTOF LC/MS analysis was carried out using an Agilent Technologies 1200 Infinity Series LC coupled with an Agilent Technologies 6540 UHD Accurate-Mass Q-TOF-LC/MS (Agilent Technologies, Santa Clara, CA, USA), equipped with an electrospray ionization Agilent Technologies Dual Jet Stream ion source (Dual AJS ESI). Chromatographic separation was achieved on a Gemini^®^ NX-C18 (3 μm, 150 × 2 mm ID) and a guard column C18 (4 × 2 mm, ID; 3μm). The mobile phase consisted of 0.1% formic acid in water milli-Q (solvent A) and methanol (solvent B) with a 25 min gradient. The mobile phase gradient (10–95% B) steps were applied as follows: 0–2 min, 10% B; 2–5 min, 70% B; 5–7 min, 80% B; 7–8 min, 90% B; maintained 4 min at 90% B; 12–16 min, 95% B; 16–18 min, 50% B; 18–22 min, and 10% B. The injection volume was 10 µL.

A mass spectrometry analysis was used with the following QTOF-MS conditions: drying gas flow (N_2_), 8.0 L min−1; nebulizer pressure, 45 psi; gas drying temperature, 370 °C; capillary voltage, 3500 V; fragmentor voltage, 130 V; skimmer voltage, 65 V; and octopole RF peak, 750 V. The Agilent Dual Jet Stream electrospray ionization (Dual AJS ESI) interface was used in the positive and negative ionization modes, and ions were acquired in the range of 100–1000 *m*/*z* for MS scans, and 50–1000 *m*/*z* for auto MS/MS scans, at a scan rate of five spectra/s for MS and three spectra/s for MS/MS, respectively. Automatic acquisition mode MS/MS was carried out using the following collision energy values: *m*/*z,* 20 eV; *m*/*z,* 30 eV and 40 eV. Internal mass correction was enabled by using two reference masses at 121.0509 and 922.0098 *m*/*z*. Instrument control and data acquisition were performed using Agilent MassHunter Workstation software B.08.00. All of the MS and MS/MS data of the validation standards were integrated by MassHunter Qualitative Analysis B.10.0 and MassHunter Quantitative Analysis B.10.0 (Agilent Technologies).

### 4.7. Risk of Dietary Exposure

Risk of dietary exposure calculation/evaluation, in the present study, consisted of measuring the presence of mycotoxins in analyzed samples. It also consisted of characterizing the distribution of one or more mycotoxin for estimating population exposure upon the consumption of average or extremely high amounts [49]. The probable daily intake (PDI, ng.kg^−1^.bw.day^−1^) of each mycotoxin through AMP consumption was estimated based on the concentration of each mycotoxin detected in the samples, the daily consumption rate of AMPs, and the average body weight of an individual consumer (70 kg). It is important to highlight that this is for genotoxic substances and substances classified as carcinogenic by the International Agency for Research on Cancer (IARC) [41] (such as AFs or OTA). EFSA, through a CONTAM Panel, considered the possibility of applying a margin of exposure (MOE) approach, with a benchmark dose lower confidence limit, for a benchmark response of 10% (BMDL10) [50]. Nonetheless, recent studies from Portugal [26] have conducted this calculation for AFs by using the TDI of 0.2 ng/kg b.w./day already proposed [51]. To characterize the risk for each mycotoxin, the PDIs were compared with the TDIs of mycotoxins established by JECFA (2001) [52] and SCF (2002) [53]. The percentage of tolerable daily intake (%TDI) from the consumption of green tea was calculated as follows: %TDI = PDI/TDI × 100.

## Figures and Tables

**Table 1:** Values of repeatability (mean recoveries of the triplicate and matrix effect ME) and sensitivity with a mix of blank AMP samples spiked at 10 times LOD.

Mycotoxin	Repeatability		Sensitivity	
	R ± SD (%)	ME ± SD ^a^ (%)	LOD (ng/g)	LOQ (ng/g)
AFB1	102 ± 1.4	9 ± 1.9	4.05	13.52
AFB2	112 ± 5.1	9.9 ± 3.8	3.30	11.00
AFG1	86 ± 0.5	13.5 ± 4.8	1.22	4.06
AFG2	97 ± 0.02	8.1 ± 2.4	2.07	6.90
OTA	92 ± 13	4.5 ± 0.96	0.93	3.11
BEA	85 ± 16	7.2 ± 0.3	0.37	1.22
ENA	100 ± 0.1	4.5 ± 0.7	0.29	0.95
ENA1	125 ± 14	3.6 ± 0.5	0.29	0.95
ENB	103 ± 4.5	4.5 ± 0.6	0.09	1.02
ENB1	112 ± 13	5.4 ± 1.2	0.52	1.74
ZEN	100 ± 2.5	3.6 ± 0.9	12.90	43.00
AOH	109 ± 0.8	4.5 ± 0.7	0.20	6.67
TENT	87 ± 6	5.4 ± 0.8	0.28	0.92
T-2	99 ± 0.01	7.2 ± 1.2	4.75	15.84
HT-2	81 ± 4.3	10.3 ± 1.3	1.59	5.30

^a^ SD: Standard deviation.

**Table 2:** MS/MS parameters for mycotoxin detection by multiple reaction monitoring (MRM).

Analyte	Rt ^a^ (min)	Parent Ion Q1 (*m*/*z*)	Product Ions Q3						DP ^c^	CEP ^c^
			Q (*m*/*z*) ^b^	CE ^c^	CXP ^c^	q (*m*/*z*) ^b^	CE^c^	CXP ^c^		
AFB_1_	7.8	313.1 [M+H]^+^	241	41	4	285	39	4	46	18
AFB_2_	7.7	315.1 [M+H]^+^	259	39	6	287	33	6	81	18
AFG_1_	7.6	329.1 [M+H]^+^	311	29	6	243	39	6	76	18
AFG_2_	7.5	331.1 [M+H]^+^	245	39	6	313	27	6	61	18
OTA	8.7	404.1 [M+H]^+^	102	97	6	239	27	6	55	21
ENA	10.3	699.4 [M+NH_4_]^+^	228	59	16	210	35	14	66	30
ENA_1_	10.1	685.4 [M+NH_4_]^+^	214	59	10	210	37	8	66	30
ENB	9.7	657.3 [M+NH_4_]^+^	214	59	10	196	39	8	51	29
ENB1	9.9	671.2 [M+NH_4_]^+^	228	57	12	214	61	10	66	29
AOH	8.5	259.0 [M+H]^+^	184	42	3	128	65	3	39	16
TENT	7.8	415.0 [M+H]^+^	256	39	2	312	29	2	55	21
BEA	10	801.2 [M+NH_4_]^+^	244	39	6	784	27	10	116	33
HT-2	8.2	442.1 [M+NH_4_]^+^	215	19	8	263	19	4	21	22
T-2	8.4	484.1 [M+NH_4_]^+^	215	29	4	185	22	4	21	23
ZEN	8.9	319.1 [M+H]^+^	282	19	4	301	15	10	26	18

^a^ Rt: Retention time; ^b^ Q: quantification transition; q: qualification transition; ^c^ de-clustering potential (DP), collision energy (CE), collision cell entrance potential (CEP), and collision cell exit potential (CXP) are all expressed in voltage.

**Table 3:** Mycotoxin incidence, mean of positive (Mp) samples, and range levels distributed according to the eight studied species of AMP varieties.

AMP	Detected Mycotoxins	Incidence (%)	Mp ± SD (ng/g)	Range (ng/g)
*Origanum vulgare (n* = 12)	AOH	9 (75)	174 ± 96	8.6–309
	ZEN	3 (25)	72 ± 29	86.6–91
*Rosmarinus officinalis (n* = 7)	AFG2	3(43)	27.7 ± 2.1	26.2–41
	ZEN	6 (86)	45 ± 21	33.7–88
	AOH	6(86)	38 ± 16	10.9–53
*Myrtus communis* (*n* = 5)	AFG1	5 (100)	6.4 ± 1.3	4.9–9
	AOH	4 (80)	40.7 ± 16	34.5–72
	TENT	5 (100)	1.7 ± 2	0.7–4.5
*Verveine officinale (n* = 4)	ENA1	1 (25)	0.3	LOD–0.3
	ENB	1 (25)	0.1	LOD–0.1
	HT-2	1 (25)	2.9	LOD-2.0
	AOH	4 (100)	199.3 ± 70	124.6–293
*Mentha spicata (n* = 2)	ZEN	2 (100)	95.7 ± 27	76.7–115
	AOH	2 (100)	138.9 ± 1.2	138.1–140
*Lavandula intermedia* (*n* = 3)	AFG1	2 (67)	7.1 ± 1.5	6–8
	ENB	3 (100)	0.2 ± 0.1	LOD–0.4
	AOH	3 (100)	53.3 ± 21	29.8–69
	TENT	2 (67)	1.6 ± 0.1	1.5–1.6
*Artemisia absinthium (n* = 2)	AOH	1 (50)	2.3	LOD–2.3
*Matricaria chamomilla (n* = 5)	AOH	5 (100)	204.6 ± 102	30.9–279

**Table 4:** Occurrence, mean levels, probable daily intake (PDI), and risk of dietary exposure of studied mycotoxins through analyzed Moroccan AMPs.

Mycotoxin	Incidence (%)	Range Levels (ng/g)	Mp ± SD ^a^ (ng/g)	Mt ± SD (ng/g)		PDI (ng/kg b.w./day) [%TDI]		TDI (ng/kg b.w./day)
				LB ^b^	UB ^c^	LB	UB	
AFG1	7 (17.5)	4.9–8.6	4.6 ± 1.4	1.16 ± 4.7	3.97 ± 1.35	-	-	-
AFG2	3 (7.5)	26.2–41.1	27.7 ± 2.1	2.42 ± 8.8	7.27 ± 8.8	-	-	-
AFs	-	-	-	-	-	-	-	-
ENA1	1 (2.5)	0.35	0.16 ± 0.3	0.019 ± 0.05	1.3 ± 0.32	-	-	-
ENB	1 (10)	LOD (0.1) ^d^	0.05 ± 0.1	0.02 ± 0.063	1.01 ± 0.06	-	-	-
ENs	-	-	-	-	-	-	-	*-*
ZEN	11 (27.5)	33.7–114.7	55.5 ± 26	18.2 ± 32.7	37.8 ± 32.7	0.67 [0.56]	1.39 [0.82]	250
AOH	34 (85)	2.3–309.5	126.2 ± 40.4	99.7 ± 97.8	116.2 ± 97	-	-	-
TENT	7 (17.5)	0.7–4.5	1.47 ± 0.8	0.29 ± 1.2	0.88 ± 0.3	-	-	-
HT-2	1 (2.5)	LOD–LOQ (2.9) ^e^	1.47 ± 2.6	0.072 ± 2.05	5.49 ± 0.5	0.003	0.203	-
T2+HT2	-	-	-	-	-	0.003	0.203	100

^a^ Mp: mean of positive samples; Mt: mean of total analyzed samples; SD: Standard deviation; ^b^ LB: Lower bound; ^c^ UB: Upper bound; ^d^ Values close to LOD; ^e^ Value between LOD and LOQ.

**Table 5:** Identification of conjugated mycotoxins and ZEA’s metabolites detected in AMP analyzed samples.

Samples Code	Mycotoxins Conjugate and Metabolites	Molecular Formula	Precursor Ion Mass (*m*/*z*)	Exact Molecular Mass (Da)	Mass Error (ppm)	Purity Score	Area	Retention Time (min)
PAM 2.d	β-ZEL	C18 H24 O5	365.1616	320.1633	1.2	82.27	273957	9.1
PAM 3.d		C18 H24 O5	379.1771	320.1623	2.3	82.95	317981	9.1
PAM 4.d		C18 H24 O5	379.1773	320.1623	1.3	98.15	312003	9.3
PAM 5.d		C18 H24 O5	319.1553	320.1615	1.62	84.71	387715	9.3
PAM 6.d		C18 H24 O5	379.1769	320.1630	0.9	98.23	326655	9.3
PAM 9.d		C18 H24 O5	379.1769	320.1630	1.5	83.70	439643	9.3
PAM 27.d		C18 H24 O5	379.1763	320.1627	2.4	98.06	599853	9.3
PAM 28.d		C18 H24 O5	365.1614	320.1631	−1.3	94.51	274979	9.2
PAM 32.d		C18 H24 O5	365.1614	320.1633	1.04	80.27	14188	9.1
PAM 8.d	α-ZEL	C18 H24 O5	319.1553	320.1629	1.3	96.50	450446	10.4
PAM 27.d		C18 H24 O5	319.1560	320.1636	1.4	97.38	185920	10.2
PAM 2.d	ZEN-14-Glc *	C24 H32 O10	525.2144	480.1992	2.5	71.67	24470	6.8
PAM 3.d		C24 H32 O10	539.2156	480.2017	2.6	69.62	20691	6.5
PAM 6.d		C24 H32 O10	525.1971	480.2028	−2.3	73.19	9149	6.6
PAM 8.d		C24 H34 O10	537.2128	481.2165	2.4	69.49	29254	6.3
PAM 8.d	β-ZEL-14-Glc *	C24 H34 O10	481.2058	482.2139	2.05	67.21	9817	15.9
PAM 8.d	ZEN-4-Sulf *	C18 H22 O8 S	399.2550	400.2624	1.1	71.02	32229	11.1
PAM 27.d		C18 H22 O8 S	399.2555	400.2625	−1.03	75.04	10390	11.1
PAM 28.d		C18 H22 O8 S	399.2539	400.2608	−2.2	92.11	11117	11.1
PAM 32.d		C18 H20 O8 S	397.0968	398.1031	−1.29	93.36	22950	13.5

* Tentative identification by Metlin Metabolites PCDL MassHunter (PCDL contains an accurate mass compound database, a collision cross section database, and an MS/MS accurate mass spectral library).

## Supplementary Materials

The following are available online at https://www.mdpi.com/2072-6651/13/2/125/s1, Figure S1: Total ion current (TIC) chromatogram from a positive sample and its extracted ion chromatogram (EIC) of β-ZEL and ZEN-14-Glc (a), scan and product ion spectrum of β-ZEL (b), and scan and product ion spectrum of ZEN-14-Glc (**c**)., Table S1: Recoveries of intraday (*n* = 3) study of spiked AMP samples and matrix.

## List of Abbreviations

AFB1	aflatoxin B1
AFB2	aflatoxin B2
AFG1	aflatoxin G1
AFG2	aflatoxin G2
AFs	aflatoxins
AMP	aromatic and medicinal plants
AOH	alternariol
BEA	beauvericin
DON-3-G	DON-3-glucoside
DON	deoxynivalenol
PDI	probable daily intake
ENA	enniatin A
ENA1	enniatin A1
ENB	enniatin B
ENB1	enniatin B1
ENs	enniatins
OTA	ochratoxin A
TENT	tentoxin
TDI	tolerable daily intake
ZEN	zearalenone
α-ZEL	α-zearalenol
β-ZEL	β-zearalenol
ZEN-4-Glc	zearalenone-4-glucoside
ZEN-4-Sulf	zearalenone-4-sulfate
α-ZEL-14-Glc	α–ZEL-14-glucoside
β-ZEL-14-Glc	β–ZEL-14-glucoside
ZEN-16-Glc	zearalenone-16-glucoside
