# Supplementary material for: Occurrence of Free and Conjugated Mycotoxins in Aromatic and Medicinal Plants and Dietary Exposure Assessment in the Moroccan Population
Source: Toxins (Basel). 2021 Feb 8;13(2):125. doi: 10.3390/toxins13020125 (PMC7915639; doi:10.3390/toxins13020125)
Supplement: Supplementary file 1 [file toxins-13-00125-s001.pdf]

Supplementary Materials: Occurrence of Free and Conjugated Mycotoxins in Aromatic and Medicinal Plants and Dietary Exposure Assessment in Moroccan Population

Aicha El Jai, Abdellah Zinedine, Ana Juan-García, Jordi Mañes, Samira Etahiri and Cristina Juan García

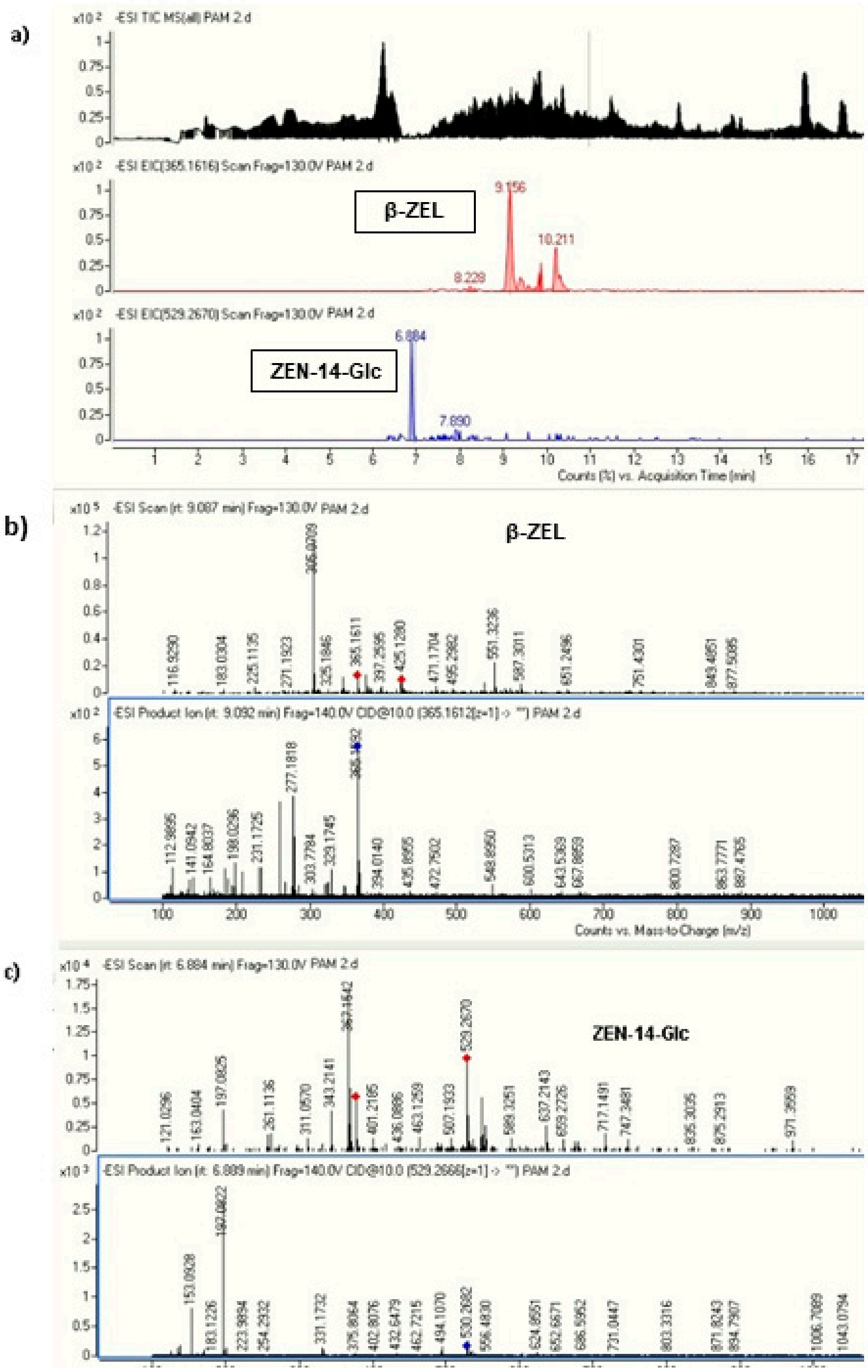

Figure S1. Total ion current (TIC) chromatogram from a positive sample and its extracted-ion chromatogram (EIC) of β-ZEL and ZEN-14-Glc (**a**), scan and product ion spectrum of β-ZEL (**b**), and scan and product ion spectrum of ZEN-14-Glc (**c**).

Table S1. Recoveries of intraday (*n* = 3) study of spiked AMP samples and matrix effect (ME) at 10 times of LOQ.

Mycotoxin	*Origanum vulgare*				*Rosmarinus officinalis*				*Matricaria chamomilla*				*Myrtus communis*				*Verveine officinale*			
	**R ± SD (%)**	**ME ± SD (%)**	**LOD (ng/g)**	**LOQ (ng/g)**	**R ± SD (%)**	**ME ± SD (%)**	**LOD (ng/g)**	**LOQ (ng/g)**	**R ± SD (%)**	**ME ± SD (%)**	**LOD (ng/g)**	**LOQ (ng/g)**	**R ± SD (%)**	**ME ± SD (%)**	**LOD (ng/g)**	**LOQ (ng/g)**	**R ± SD (%)**	**ME ± SD (%)**	**LOD (ng/g)**	**LOQ (ng/g)**
AFB1	101± 1.42	10 ±2	4.50	15	70.7± 1	7 ± 1.40	3.15	10.51	91±1.28	9.01 ± 1.80	4.05	13.52	90 ± 1.26	8.9 ± 1.78	4.01	13.36	111±1.56	11 ± 2.2	4.95	16.53
AFB2	109±5.14	11 ± 4	3.66	12.2	76.3± 3.60	8 ± 2.80	2.56	8.55	98± 4.63	9.9 ± 3.60	3.30	11.00	97 ± 4.58	9.8 ± 3.56	3.26	10.87	120 ± 5.66	12 ± 4.4	4.03	13.44
AFG1	96±0.51	15 ± 5	1.35	4.51	67.2± 0.40	10 ± 3.50	0.95	3.16	86± 0.46	13.5 ± 4.50	1.22	4.06	85 ± 0.45	13.3 ± 4.4	1.20	4.01	106 ± 0.56	16.5 ±5.5	1.49	4.97
AFG2	99±0.20	9 ± 2.50	2.30	7.66	69.3± 0.10	6 ± 1.75	1.61	5.36	89±0.18	8.1 ± 2.25	2.07	6.90	88 ± 0.18	8 ± 2.30	2.05	6.82	109 ± 0.22	9.9 ± 2.7	2.53	8.43
OTA	95± 12.90	5 ± 1	1.03	3.45	66.5±9	4 ± 0.70	0.72	2.42	86± 11.60	4.5 ± 0.90	0.93	3.11	84 ± 11.46	4.4 ± 0.89	0.92	3.07	105 ± 14.2	5.5 ± 1.1	1.13	3.80
BEA	88±16.30	8 ± 0.30	0.41	1.35	61.6± 11.4	6 ± 0.21	0.29	0.95	79± 14.70	7.2 ± 0.27	0.37	1.22	78 ± 14.5	7.1 ± 0.30	0.36	1.20	97 ± 17.94	8.8 ± 0.33	0.45	1.49
ENN A	102±0.10	5 ± 0.70	0.32	1.06	71.4±0.10	4 ± 0.49	0.22	0.74	92 ± 0.10	4.5 ± 0.63	0.29	0.96	91 ± 0.10	4.4 ± 0.62	0.28	0.94	112 ± 0.12	5 ± 0.77	0.35	1.17
ENN A1	121±14.20	4 ± 0.50	0.32	1.06	84.7± 9.90	2.8 ± 0.35	0.22	0.74	109 ± 12.80	3.6 ± 0.45	0.29	0.96	108 ± 12.6	3.6 ± 0.45	0.28	0.94	133 ± 15.61	4.5 ± 0.55	0.35	1.17
ENN B	108± 4.50	5 ± 0.60	0.10	1.13	75.6±3.10	3.5 ± 0.42	0.07	0.79	97 ± 4.03	4.5 ± 0.54	0.09	1.02	96 ± 3.98	4.5 ± 0.53	0.09	1.01	119 ± 4.92	5.4 ± 0.66	0.11	1.24
ENN B1	111± 13	6 ± 1.20	0.58	1.93	77.7± 9.10	4.2 ± 0.84	0.41	1.35	100 ± 11.70	5.4 ± 1.08	0.52	1.74	99 ± 11.57	5.4 ± 1.07	0.52	1.72	122 ± 14.31	6.7 ± 1.32	0.64	2.12
ZEN	101± 2.50	4 ± 0.89	14.32	47.7	70.7± 0.50	2.8 ± 0.62	10.02	33.42	91 ± 2.25	3.6 ± 0.80	12.90	43.01	90 ± 2.23	3.6 ± 0.79	12.75	42.49	111 ± 2.75	4.3 ± 0.98	15.77	52.6
AOH	107± 0.75	5 ± 0.75	0.22	7.4	74.9± 0.53	3.5 ± 0.53	0.15	5.18	96 ± 0.68	4.5 ± 0.68	0.20	6.67	95 ± 0.67	4.5 ± 0.67	0.20	6.59	118 ± 0.83	5.4 ± 0.83	0.24	8.15
TENT	85± 5.88	6 ± 0.78	0.31	1.02	59.5± 4.12	4.2 ± 0.55	0.22	0.71	76 ± 5.30	5.4 ± 0.7	0.28	0.92	76 ± 5.23	5.3 ± 0.69	0.28	0.91	94 ± 6.47	6.4 ± 0.86	0.34	1.12
T-2	95± 1.20	8 ± 1.20	5.27	17.6	66.5±0.84	5.6 ± 0.84	3.69	12.31	86 ± 1.08	7.2 ± 1.08	4.75	15.84	85 ± 1.07	7.1 ± 1.07	4.69	15.65	105 ± 1.32	8.7 ± 1.32	5.80	19.40
HT-2	80± 4.30	11 ± 1.40	1.69	5.63	56± 2.98	7.7 ± 0.98	1.18	3.94	72 ± 3.84	9.9 ± 1.26	1.52	5.07	79 ± 3.79	10.9 ± 1.20	1.67	5.57	96± 4.69	13.2 ± 1.54	2.03	6.80

	Mentha spicata				*Lavandula Intermedia*				*Artemisia absinthium*				*MIX*			
**Mycotoxin**	**R ± SD (%)**	**ME ± SD (%)**	**LOD (ng/g)**	**LOQ (ng/g)**	**R ± SD (%)**	**ME ± SD (%)**	**LOD (ng/g)**	**LOQ (ng/g)**	**R ± SD (%)**	**ME ± SD (%)**	**LOD (ng/g)**	**LOQ (ng/g)**	**R ± SD (%)**	**ME ± SD (%)**	**LOD (ng/g)**	**LOQ (ng/g)**
AFB1	88 ± 1.28	8.6 ± 1.8	3.90	13.01	108 ± 1.70	10.7 ± 2.40	4.81	16.03	68 ± 1.38	6.8 ± 1.94	3.06	10.19	102 ± 1.42	9 ± 1.92	4.05	13.52
AFB2	95 ± 4.62	9.5 ± 3.59	3.17	10.59	116 ± 6.17	11.7 ± 4.80	3.91	13.04	74 ± 4.99	7.5 ± 3.88	2.49	8.29	112 ± 5.14	9.9 ± 3.83	3.30	11.00
AFG1	83 ± 0.43	13 ± 4.5	1.17	3.91	102 ± 0.61	16 ± 6	1.44	4.82	65 ± 0.49	10.2 ± 4.85	0.92	3.06	86 ± 0.51	13.5 ± 4.79	1.22	4.06
AFG2	86 ± 0.18	7.8 ± 2.3	1.99	6.64	106 ± 0.24	9.6 ± 3	2.46	8.18	67 ± 0.19	6.1 ± 2.43	1.56	5.20	97 ± 0.02	8.1 ± 2.39	2.07	6.90
OTA	82 ± 11.57	4.3 ± 0.9	0.89	2.99	101 ± 15.46	5.3 ± 1.20	1.10	3.69	65 ± 12.50	3.4 ± 0.97	0.70	2.34	92 ± 12.88	4.5 ± 0.96	0.93	3.11
BEA	76 ± 14.67	6.9 ± 0.27	0.36	1.17	94 ± 19.60	8.5 ± 0.36	0.44	1.44	60 ± 15.80	5.4 ± 0.29	0.28	0.92	85 ± 16.29	7.2 ± 0.29	0.37	1.22
ENN A	88 ± 0.10	4.3 ± 0.63	0.28	0.92	109 ± 0.13	5.3 ± 0.84	0.34	1.13	69 ± 0.11	3.4 ± 0.68	0.22	0.72	100 ± 0.11	4.5 ± 0.67	0.29	0.95
ENN A1	105 ± 12.70	3.5 ± 0.45	0.28	0.92	129 ± 17.02	4. 3 ± 0.60	0.34	1.13	82 ± 13.70	2.7 ± 0.49	0.22	0.72	125 ± 14.18	3.6 ± 0.48	0.29	0.95
ENN B	94 ± 4	4.3 ± 0.54	0.09	0.98	115 ± 5.36	5.3 ± 0.72	0.11	1.21	73 ± 4.34	3.4 ± 0.58	0.07	0.77	103 ± 4.47	4.5 ± 0.57	0.09	1.02
ENN B1	96 ± 11.70	5.2 ± 1.08	0.50	1.67	119 ± 15.60	6.4 ± 1.44	0.62	2.06	75 ± 12.60	4.1 ± 1.16	0.39	1.31	112 ± 13.00	5.4 ± 1.15	0.52	1.74
ZEN	88 ± 2.30	3.5 ± 0.8	12.42	41.39	108 ± 3	4.3 ± 1.07	15.30	50.99	69 ± 2.43	2.7 ± 0.86	9.72	32.42	100 ± 2.50	3.6 ± 0.85	12.90	43.00
AOH	93 ± 0.70	4.3 ± 0.67	0.19	6.42	114 ± 0.90	5.3 ± 0.90	0.23	7.90	73 ± 0.73	3.4 ± 0.73	0.15	5.02	109 ± 0.75	4.5 ± 0.72	0.20	6.67
TENT	74 ± 5.30	5.2 ± 0.7	0.27	0.88	91 ± 7.06	6.4 ± 0.94	0.33	1.09	58 ± 5.70	4.1 ± 0.76	0.21	0.69	87 ± 5.88	5.4 ± 0.75	0.28	0.92
T-2	82 ± 1.08	6.9 ± 1.08	4.57	15.24	101 ± 1.44	8.5 ± 1.44	5.63	18.78	65 ± 1.16	5.4 ± 1.16	3.58	11.94	99 ± 0.01	7.2 ± 1.15	4.75	15.84
HT-2	69 ± 3.80	9.5 ± 1.26	1.47	4.88	95 ± 5.11	13.1 ± 1.68	2.01	6.69	54 ± 4.13	7.5 ± 1.36	1.15	3.82	81 ± 4.26	10.3 ± 1.34	1.59	5.30
